# Microvascular ultrastructural changes precede cognitive impairment in the murine APPswe/PS1dE9 model of Alzheimer’s disease

**DOI:** 10.1007/s10456-017-9568-3

**Published:** 2017-07-25

**Authors:** Patricia Kelly, Paul Denver, Simon C. Satchell, Maximilian Ackermann, Moritz A. Konerding, Christopher A. Mitchell

**Affiliations:** 1 School of Biomedical Sciences, University of Ulster, Coleraine, Northern Ireland, UK; 2 Dorothy Hodgkin Building, University of Bristol, Bristol, UK; 3 Institute of Functional and Clinical Anatomy, University Medical Centre, Johannes Gutenberg-University Mainz, Mainz, Germany

**Keywords:** Alzheimer’s disease, APP/PS1 mice, Microvascular corrosion casting, Scanning electron microscopy

## Abstract

Cerebral and systemic organ microvascular pathologies coexist with human Alzheimer’s disease (AD) neuropathology. In this study, we hypothesised that both cerebral and systemic microvascular pathologies exist in 4- to 5-month-old male APPswe/PS1dE9 (APP/PS1) transgenic mice prior to the onset of cognitive impairment. To assess this we examined recognition memory in both wild-type and APP/PS1 mice using the object recognition task (ORT; *n* = 11 per group) and counted thioflavin-S-positive plaques in brain (*n* = 6 per group). Vascular casts of brain, liver, spleen and kidneys were examined using scanning electron microscopy (*n* = 6 per group), and the urinary albumin-to-creatinine ratio (uACR; *n* = 5 per group) was measured as an index of glomerular permeability. Murine recognition memory was intact, as demonstrated by a significant preference for the novel object in the ORT paradigm. Brain sections of wild-type mice were devoid of thioflavin-S positivity, whereas age-matched APP/PS1 mice had an average of 0.88 ± 0.22 thioflavin-S-positive plaques in the cortex, 0.42 ± 0.17 plaques in the dentate gyrus and 0.30 ± 0.07 plaques in the cornus ammonis 1 region. The profiles of casted cerebral capillaries of wild-type mice were smooth and regular in contrast to those of APP/PS1 mice which demonstrate characteristic (0.5–4.6 μm) ‘tags’. APP/PS1 mice also had a significantly reduced hepatic vessel number (*p* = 0.0002) and an increase in the number of splenic microvascular pillars (*p* = 0.0231), in the absence of changes in either splenic microvascular density (*p* = 0.3746) or glomerular ultrastructure. The highly significant reduction in uACR in APP/PS1 mice compared to wild-type (*p* = 0.0079) is consistent with glomerular microvascular dysfunction. These findings highlight early microvascular pathologies in 4- to 5-month-old APP/PS1 transgenic mice and may indicate an amenable target for pharmacological intervention in AD.

## Introduction

Dementia is characterised by heterogeneous pathologies associated with neurodegenerative lesions that progressively affect multiple cognitive domains and irreversibly impair the ability to conduct the tasks required for independent living [1]. Recent global estimates indicate that 46 million individuals have dementia and with 10 million new cases of dementia occurring each year, it is expected that by the year 2018 the global cost of dementia will exceed 1 trillion dollars [2]. AD is the most common form of dementia, which accounts for 60–80% of all dementia cases in the general population [2]. As the insidious pathological pathways to neurodegeneration remain elusive and the clinically used neurotransmitter-based therapies are ineffective in altering disease course, it is expected that by the year 2050 there will be 106.8 million individuals with AD worldwide [3–6], highlighting the urgent need to identify causative pathology.

The proteinaceous accumulations characteristic of AD includes parenchymal amyloid plaques and intraneuronal tau which were first formally described by Dr. Aloysius ‘Alois’ Alzheimer in 1906 following a post-mortem examination of 51-year-old Auguste Deter, who presented with ante-mortem ‘delirium’ [7]. Dr. Alzheimer observed concomitant cerebrovascular abnormalities that included atherosclerotic changes to large cerebral vessels, a complete absence of infiltrating vessels and an excessive proliferation of the endothelium [7]. Cerebrovascular lesions such as infarctions, lacunas and small vessel disease are concomitant with AD neuropathology, which along with amyloid plaques and neurofibrillary tangles (NFTs) additively lower the threshold for cognitive impairment [8–13]. Clinicopathological findings show that a similar magnitude of cognitive dysfunction can be elicited by lower cerebral densities of amyloid plaques and NFTs coinciding with cerebrovascular lesions compared to a higher cerebral burden of amyloid plaques and NFTs alone [12, 14]. An ante-mortem aggregation of the vascular-based risk factors for AD that include type-2 diabetes mellitus (T2DM), hypertension, hypercholesterolemia and obesity is associated with an increased prevalence of cerebrovascular lesions at post-mortem [15].

Pathological alterations to the integrity of the cerebrovasculature may critically alter the function of the neurovascular unit (NVU) and cause neuronal injury via an impairment of cerebral perfusion, an unrestricted brain entry of circulatory compounds, reduced cerebral amyloid clearance and vascular and parenchymal accumulations of cerebrotoxins [13, 16, 17]. Quantitative analysis of the blood-to-brain transfer of a gadolinium contrast agent in human participants using a high-resolution magnetic resonance imaging (MRI) protocol showed an age-dependent and region-specific disruption of the blood–brain barrier (BBB) in the hippocampus, a brain region critical for learning and memory [18]. Interestingly, the aforementioned study showed that hippocampal BBB disruption was greater in individuals with mild cognitive impairment and independent of hippocampal atrophy, demonstrating an early role for BBB dysfunction in the pathogenesis of AD [18]. A recent comprehensive data-driven analysis of over 7700 multiple-modality brain images, plasma and cerebrospinal fluid biomarkers from 1171 human subjects from the Alzheimer’s Disease Neuroimaging Initiative (ADNI) proposed that vascular dysfunction precedes amyloid plaque deposition in the pathological cascade of AD [19].

The findings of animal studies also support the proposal that pathological changes to the vasculature are an early event in the pathogenesis of AD. The Tg2576 transgenic murine AD model harbouring the amyloid precursor protein (APP) 695 isoform with the double Swedish mutation [20] exhibits cerebral hypoperfusion from 2 to 3 months of age, which precedes the onset of amyloid plaques that accumulate in the brain of Tg2576 mice from 11 to 13 months of age [21]. APP23 transgenic mice carrying the APP751 isoform with a double Swedish mutation [22] show multiple areas of vessel elimination in the cortex at 4–5 months of age, which is prior to the onset of amyloid plaques at 6 months of age [23]. The aforementioned study used the microvascular corrosion casting technique to recreate the three-dimensional structure of cerebral vessels using a polymerising resin solution followed by a scanning electron microscopic examination of the vascular ultrastructure [23, 24]. Interestingly, an additional study of APP23 mice that examined cerebral hemodynamics using magnetic resonance angiography prior to microvascular corrosion casting showed that areas of flow voids on the angiograms occupied the same brain regions as the vascular structural deformations in the resin cerebral vascular casts that were examined by scanning electron microscopy (SEM; [25]).

In the present study, we use the microvascular corrosion casting technique with SEM to recreate and examine the fine structure of small calibre vessels of the APPswe/PS1dE9 (APP/PS1) mouse model of AD. APP/PS1 mice carry the “Swedish mutation” (APP695 isoform) in addition to an exon-9 deleted variant of presenilin-1 (PS1) within independent vectors, which results in over-expression of these proteins primarily in the brain and heart [26, 27]. APP/PS1 mice are reported to progressively accumulate parenchymal amyloid plaques by 4–5 months [28], develop cerebral amyloid angiopathy (CAA) from 6 months [28] and exhibit cognitive deficits in behavioural tasks from 7 months of age [29, 30]. Accordingly, we have examined both the cerebral and systemic vascular ultrastructure of young 4- to 5-month-old male APP/PS1 mice at the age when cerebral amyloid plaques begin to accumulate, prior to development of both CAA and cognitive deficits. We sought to determine whether disturbances of microvascular structure and function are a systemic phenomenon (rather than being CNS specific) in the APP/PS1 mouse by examining organs previously shown by I^125^-labelling studies to be associated with the metabolism of β-amyloid, namely kidney, spleen and liver. Accordingly, we assessed urinary albumin-to-creatinine ratios (uACR) as a measure of glomerular microvascular function in young 4- to 5-month-old APP/PS1 mice. Measuring renal dysfunction in AD is receiving attention as a potential clinical marker, with a cross-sectional study of individuals with AD identifying a high proportion with altered kidney function [33]. Furthermore, a longitudinal study showed that men >75 years of age with elevated uACR are more likely to show cognitive decline over a 6-year period [34]. In addition, we have shown that 9-month-old male APP/PS1 mice demonstrate significant splenic microvascular loss in addition to ultrastructural abnormalities in glomerular capillaries compared to age-matched, wild-type littermates [31]. Most significantly, 9-month-old APP/PS1 mice exhibit numerous cerebral cortical microvascular microaneurysms and widespread extravasation of resin casting solution from cerebral capillaries [31] that coexists with cerebral amyloid plaques, CAA [28] and cognitive deficits [32]. In the present study, we test the hypothesis that in 4- to 5-month-old APP/PS1 mice pathological changes to the cerebral, hepatic, splenic and renal microvasculature precede the onset of measurable cognitive impairment.

## Materials and methods

### Animals

Male APP/PS1 transgenic mice on a C57BL/6 genetic background (The Jackson Laboratory, Bar Harbour, Maine, USA) were mated with female C57BL/6 mice (Harlan, Blackthorn, UK) within a specialised facility with food and water available ad libitum. The temperature within the facility was maintained at 21.5 ± 1 °C and an automated lighting system created 12-h light/12-h dark cycles. Pups aged between 21 and 28 days old were removed from their parental cage with genotyping performed by collection of an ear biopsy. DNA extracted from the ear biopsy was amplified using polymerase chain reaction (PCR) with primers specific for the APP sequence. All genotyped animals were housed in same sex social groups of up to three littermates per cage. At 4–5 months of age, the mice within the wild-type and APP/PS1 groups were listed in chronological order of date of birth and each mouse was assigned a number in sequential order. Random numbers were generated using the ‘RANDBETWEEN(1, total number of mice within group) function (Microsoft^®^ Excel, Redmond, WA, USA), and mice with an assigned number matching the random number were assigned into experimental groups and caged individually. Experimental procedures were in accordance with Animals (Scientific Procedures) Act, UK of 1986.

### Open field task

The individual spontaneous locomotion and exploratory behaviour of 4- to 5-month-old male wild-type and APP/PS1 mice (*n* = 11 per group) within an open field arena was recorded and quantitatively assessed. Mice were selected for the experimental procedure in a random manner and to avoid experimenter bias, their genotype was concealed by a code that was assigned by an assistant. At 1 week prior to the behavioural task, the investigator handled the animals individually within the testing room for 3 min each day for 7 days, in a bid to acclimatise the animals to the investigator and to the testing room. The open field task (OFT) consisted of a circular arena of 58 cm in diameter with walls that were 31 cm high. The arena was dimly illuminated by a 60 W lamp. Each animal explored the open field arena for 5 min, and murine behaviour was recorded by a computerised tracking system and camera (Biosignals, New York, USA), positioned at a distance of 2 m above the arena. Path length, linearity, the number of rearing events (each instance that the mouse elevated its front paws), the number of faecal pellets produced within the arena and the exploration of the centre of the arena versus the periphery by each of the 4- to 5-month-old wild-type and APP/PS1 mice were determined. The open field arena was cleaned thoroughly using 70% ethanol in a bid to reduce olfactory cues prior to introducing the next mouse under test to the open field arena.

### Novel object recognition task

Recognition memory, a form of working memory [35], was evaluated in 4- to 5-month-old male wild-type and APP/PS1 mice using the novel object recognition task (ORT). The novel ORT paradigm examines the integrity of neural processes required for the storage and recall of a previously explored ‘familiar object’ motivating exploration of a ‘novel object’, without need for negative reinforcement [36]. At 24 h following completion of the OFT, each animal was in turn individually placed into the open field arena for 10 min for the acquisition phase of the novel ORT task. The open field arena contained two randomised objects (white balls or red cubes), secured to the floor at a distance of 15 cm from either wall. The exploration of either object, defined as the amount of time that an animal positioned its snout ≤2 cm from the object, was recorded. Immediately upon completion of the acquisition phase, the animal was returned to its’ home cage for a retention period of 3 h prior to being placed into the arena for the test phase of the task. The test phase of the novel ORT paradigm consisted of an individual exploration of the arena by each animal for 10 min. The arena contained one object previously encountered by the animal during the acquisition phase of the task and one ‘novel’ object (white ball or red cube) that the animal had not previously explored. Murine exploration of both objects was recorded in the same manner as in the acquisition phase. The recognition index (RI), defined as the amount of time that each mouse spent exploring either object (tA or tB) divided by the amount of time spent exploring both objects (tA or tB/tA + tB) × 100, was determined for each animal in both the acquisition phase and test phase of the task.

### Quantitative determination of thioflavin-S-positive amyloid plaques in murine brain

The number of thioflavin-S-positive plaques within the cortex and the dentate gyrus and cornus ammonis 1 (CA1) sub-regions of the hippocampus were identified histologically and counted in a blinded manner in 4- to 5-month-old male wild-type and APP/PS1 mice (*n* = 6 per group) that were randomly assigned to the experimental procedure. Each animal received an intraperitoneal injection of heparin [Sigma-Aldrich Ltd, Dorset, UK (180 IU/kg bodyweight (bw))] dissolved in sterile saline solution (0.9% w/v NaCl) at a minimum of 15 min prior to inhalation exposure to isoflurane (Isoflo Inhalation 100% w/v Vapour, liquid isoflurane, Abbott Laboratories Ltd, Berkshire, UK). The animal subsequently received an intraperitoneal injection of 0.3 mL Pentobarbital sodium (Dolethal 200 mg/mL solution for injection, Vetoquinol UK Ltd, Buckingham, UK) and an adequate level of anaesthesia was confirmed by loss of the pedal withdrawal reflexes. The murine heart was exposed surgically, and the left ventricle was carefully cannulated with a cut made to the right atrium. The entire murine circulatory system was flushed with 20 mL phosphate-buffered saline [(PBS) Oxoid Ltd, Hampshire, UK] solution heated to 37 °C followed by 20 mL filtered 4% paraformaldehyde (PFA) solution [37] heated to 37 °C. The murine brain was excised from the cranial vault by careful dissection and stored overnight in 4% PFA solution prior to transfer to a solution of 30% sucrose (Sigma-Aldrich Ltd, Dorset, UK) in PBS solution for up to 5 days. Each whole brain specimen was mounted in an upright position onto a 25-mm stainless steel chuck (Leica Biosystems, Milton Keynes, UK) containing Tissue-Tek OCT compound (Sakura Finetek, AJ Alphen aan den Rijn, The Netherlands) and subsequently cooled in 2-methylbutane (Sigma-Aldrich Ltd, Dorset, UK) on dry ice. Each whole brain specimen was trimmed using a cryostat (Leica CM1850, Leica Biosystems, Milton Keynes, UK) to approximately bregma −0.94 mm [38], and the first 20-µm coronal whole brain section was collected using a cooled paint brush with careful transfer to a cryoprotectant buffer (30% ethylene glycol, 25% glycerine in PBS solution). Every seventh whole brain section was collected thereafter until a depth of bregma −2.92 mm [38]. Eight whole brain sections from each animal were washed 3 times in dd.H_2_O and mounted onto a glass microscope slide, pre-coated with 2% v/v aminopropyltriethoxysilane (Sigma-Aldrich, Dorset, UK) in acetone (Sigma-Aldrich Ltd, Dorset, UK). Each slide was placed within an increasing concentration of 70–80% ethanol (Sigma-Aldrich Ltd, Dorset, UK) for 1 min prior to being placed into a solution of filtered 1% thioflavin-S in 80% ethanol for 15 min. The slides were then transferred into a decreasing gradient of 80–70% ethanol (Sigma-Aldrich Ltd, Dorset, UK) for 1 min and dd.H_2_O for 1 min [39]. Each slide was dried in a light-protected container for at least 2 h and then cover-slipped using aqueous mounting media (Vector mounting medium, Vector Laboratories, California, USA). Blinding of the APP/PS1 status for each slide was performed using a pre-assigned code provided by an assistant. Two areas within each cortex, dentate gyrus and CA1 region from both hemispheres of each whole brain section were selected at random, imaged using a 10× objective magnification (Axio Scope A1, Zeiss, Germany) and stored prior to blinded analysis. A single, randomly placed, counting frame (1050 µm × 1400 µm) was super-imposed on each of the cortical and hippocampal regions examined and the total numbers of plaques identified and counted using ImageJ software. GraphPad prism software was then used to determine the average number of thioflavin-S-positive plaques for each brain region per animal. Data are presented as mean ± SEM for each brain region.

### Microvascular corrosion casting

Male wild-type and APP/PS1 mice aged 4–5 months of age (*n* = 6 per group) were randomly assigned to the experimental procedure. Each animal received an intraperitoneal injection of heparin [(2500 IU/L) in 0.9% w/v NaCl; Ratiopharm, Gmbh] at a minimum of 15 min prior to the administration of non-recovery anaesthesia [100 mg/kg bw ketamine (Ketavet, Pfizer, Berlin, Germany); 16 mg/kg bw xylazine (Rompun, Bayer, Leverkusen, Germany)]. Once an adequate plane of anaesthesia was confirmed by loss of pedal withdrawal reflexes, the animal was secured in a supine position under a stereomicroscope [Leica MZ6; Leica Microsystems (UK) Ltd; Milton Keynes, UK]. The murine heart was surgically exposed and a cannula (Acufirm, 1428LL) carefully inserted into the aorta and secured by the tying of a silk suture around the aorta. The murine right atrium was cut immediately prior to the perfusion of 15 mL saline solution [(0.9% w/v NaCl) heated to 37 °C)], followed by 15 mL [(2.5% glutaraldehyde in 0.1 M phosphate buffer pH 7.4 (Agar Scientific, Essex, UK)] heated to 37 °C, with subsequent perfusion of 15 mL PU4ii casting solution (VasQtec, University of Zurich, Switzerland), prepared as directed by the manufacturer. The casted mice remained in a supine position at room temperature for 12 h prior to the careful removal of the brain, liver, spleen and kidneys from each animal. Each resin-casted whole brain and kidney specimen was bisected, and the left lobe was carefully removed from each casted liver specimen.

### Scanning electron microscopic evaluation of cerebral, hepatic, splenic and renal vasculature

The casted brain, left hepatic lobe, spleen and kidney specimens were individually placed into a macerating solution of 5–7.5% potassium hydroxide solution (Sigma-Aldrich Company Ltd, Dorset, UK). The macerating solution was renewed daily until all of the tissue that surrounded the casted vasculature had been removed. Each resin-casted specimen was carefully washed using dd.H_2_O and placed into a dust-protected container, lined with fine filter paper. Each specimen air-dried for 72 h [24] prior to being secured onto an aluminium stub and homogeneously coated with a thin layer of palladium and gold (E5100; Quorum Technologies Ltd, East Sussex, UK) for SEM examination.

### Quantitative analysis of cerebral, hepatic and splenic microvascular ultrastructure

The identity of each resin-casted murine brain was concealed using a code that was assigned by an assistant. Areas of cortical vasculature within each casted brain specimen were selected at random by an assistant and imaged at 500× using a SEM (Philips XL30, Eindhoven, Netherlands), at an acceleration voltage of 15 keV. The generated micrographs were spatially calibrated using ImageJ software (US National Institutes of Health, Bethesda, Maryland, USA). Ten cortical capillaries (that were not obscured by other vessels) were selected, and their lengths as well as diameters measured using ImageJ software. Five areas of hepatic and splenic microvasculature were selected at random by an assistant and imaged at 800× and 1400×, respectively, using a SEM (ESEM, FEI Quanta 200 FEG, Holland FEI Company) at an acceleration voltage of 10–15 keV. The generated micrographs had their identities concealed with a number, provided by an assistant. The number of hepatic sinusoidal vessels not concealed by other vessels within a counting frame (dimensions of 330 μm × 380 μm) recorded. The area occupied by splenic microvasculature within the spatially calibrated micrographs (counting frame with dimensions of 190 μm × 220 μm) was determined using ImageJ software. The number of splenic intussusceptive pillars (for details see [31]) was counted within each of the coded micrographs.

### Determining the uACR in collected mouse urine

Male wild-type and APP/PS1 mice aged 4–5 months (*n* = 5 per group) were randomised into the experimental procedure and 200 µL urine collected directly from each animal. The collected urine was then transferred into a sterilised collection tube that subsequently had its identity concealed by a code assigned by an assistant. The concentration of albumin within each of the coded urine samples was determined using a Mouse Albumin ELISA (Mouse Albumin ELISA Quantitation Set, Bethyl Laboratories Inc, Montgomery, USA) and the concentration of creatinine determined using an enzymatic assay (Thermo Scientific) on a Konelab Clinical Analyser at the Langford Diagnostic Laboratory, University of Bristol, UK.

### Statistical analyses

The mean values of independent variables were statistically compared using GraphPad Prism 6 Software (GraphPad Software Inc, California, USA). Normality of the distribution was assessed using a Kolmogorov-Smirnov assessment. Data sets with a *p* value <0.05 were deemed to be significantly different from normal and were assessed nonparametrically using the Mann–Whitney *U* test. Data sets that were deemed to be normal were assessed parametrically using either an analysis of variance (ANOVA) or *t* test. Data were considered to be statistically different when *p* value <0.05. The data are presented in graphs generated using GraphPad Prism software and values shown ±standard error of the mean.

## Results

### Exploratory behaviour of 4- to 5-month-old APP/PS1 mice is not significantly different from age-matched wild-type mice

**Fig. 1:**
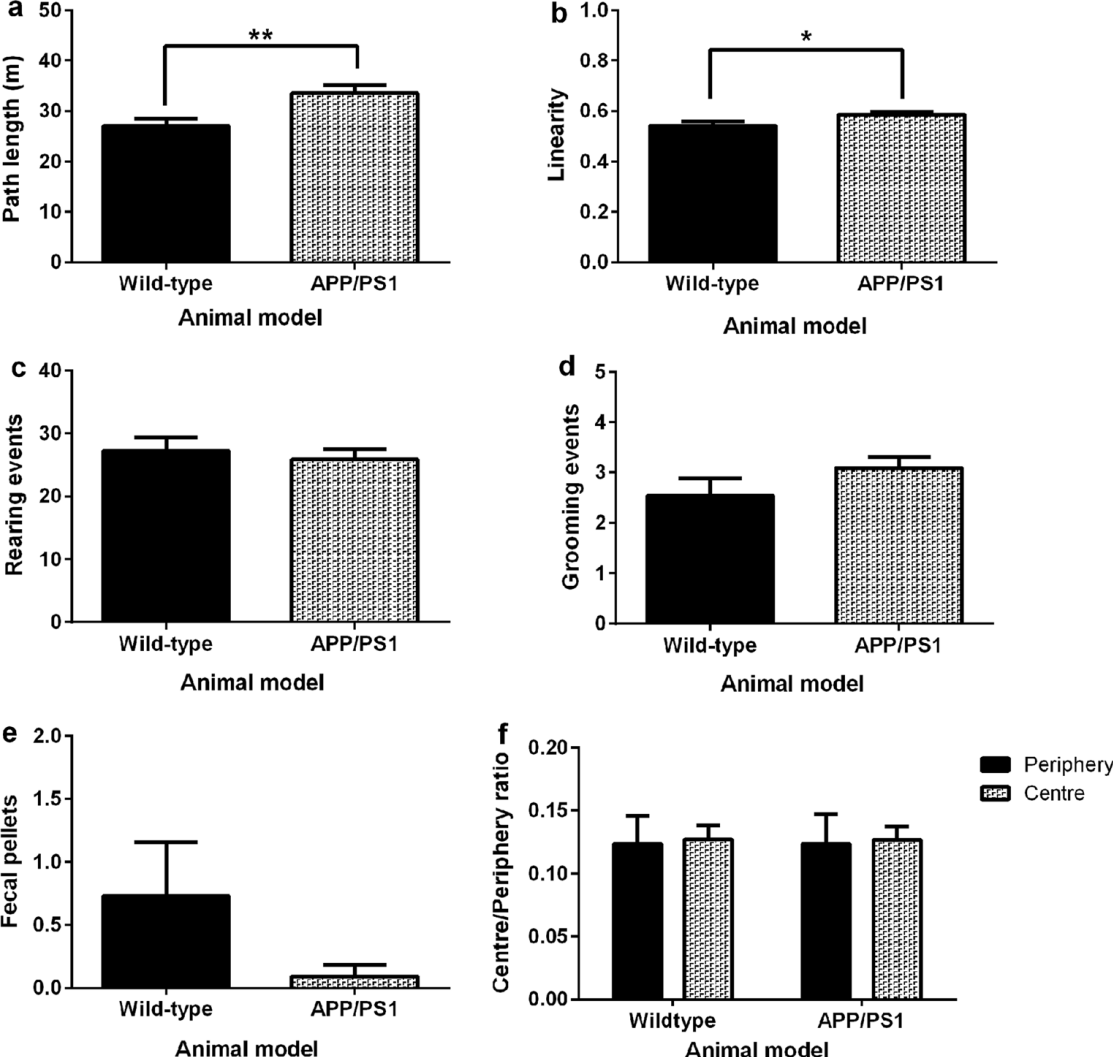
OFT assessed the spontaneous behaviour of 4- to 5-month-old wild-type and APP/PS1 transgenic mice during an individual exploration of a circular arena (*n* = 11 per group). The locomotor activity of wild-type mice within the arena was significantly lower than age-matched APP/PS1 mice which exhibited increased path length (**p = 0.0069; **a**) and linearity (*p = 0.0308; **b**). There were no statistical differences in either the number of rearing events (*p* = 0.6285; **c**), grooming events (*p* = 0.1883; **d**) or faecal pellets (*p* = 0.1617; **e**) produced by wild-type mice when compared to APP/PS1 mice. Mice spent an equal amount of time exploring the periphery and the centre of the arena (**f**). Statistical analysis was conducted using the unpaired *t* test (**a**–**e**) and two-way ANOVA with Bonferroni’s multiple comparison test (**f**) and presented as mean ± standard error of the mean (SEM)

Murine spontaneous locomotion and exploratory behaviour was assessed within a dimly illuminated open field arena. Wild-type mice had a shorter path length (**p = 0.0069; Fig. 1a) and path linearity (*p = 0.0308; Fig. 1b) when compared to age-matched APP/PS1 mice. There was no significant difference in the number of rearing events exhibited by wild-type mice when compared to age-matched APP/PS1 mice (*p* = 0.6285; Fig. 1c). Similarly, the average number of grooming events (*p* = 0.1883; Fig. 1d), faecal pellets produced (*p* = 0.1617; Fig. 1e) and the ratio of exploration of the periphery of the arena compared to the centre (*p* > 0.05; Fig. 1f) were not significantly different between groups.

### Working memory is intact in 4- to 5-month-old wild-type and APP/PS1 mice

**Fig. 2:**
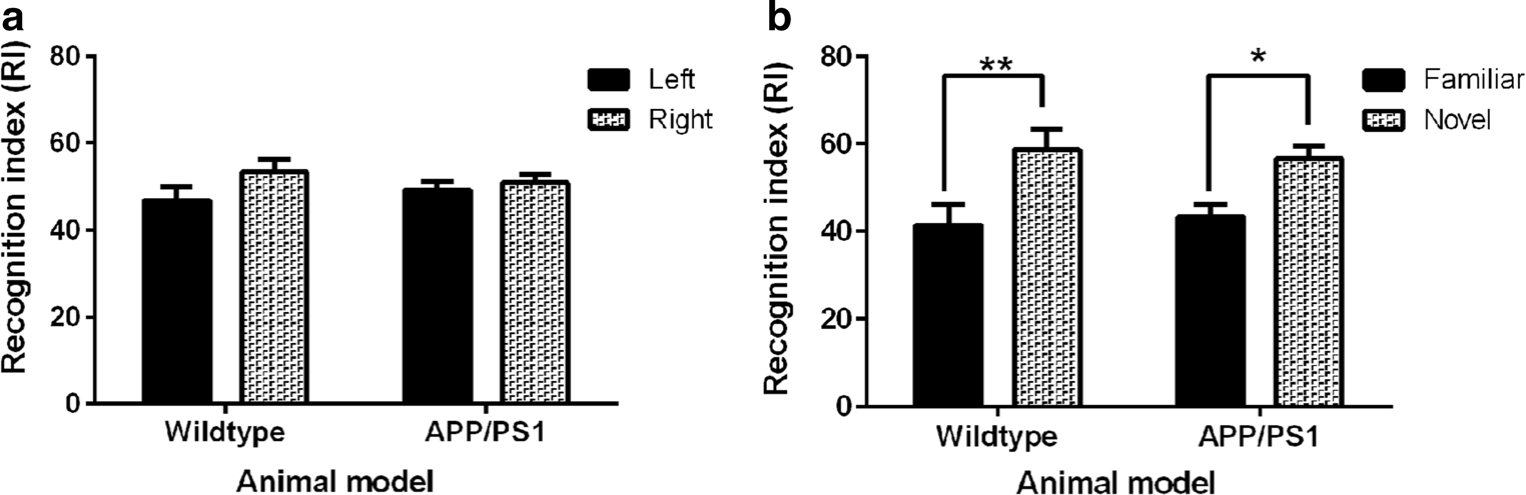
Recognition memory of 4- to 5-month-old wild-type and APP/PS1 transgenic mice (*n* = 11 per group) was quantitatively determined within an open arena using the novel ORT paradigm. In the acquisition phase of the behavioural task **a** the recognition indices (RI) were not significantly different for either wild-type or APP/PS1 transgenic mice thus showing similar exploration of either object within the open arena. After a 3-h time delay between the acquisition and test phase when mice were reintroduced into the arena and exposed to the familiar and novel object the RI was significantly greater for the novel object demonstrating a significantly greater exploration of novelty by both groups of mice (*p* < 0.05; **b**). Statistical analysis was conducted using two-way ANOVA with Bonferroni’s multiple comparison test and data is presented as mean ± SEM

The recognition memory of 4- to 5-month-old wild-type and APP/PS1 mice was assessed using the novel ORT paradigm [40; Fig. 2]. There was no significant difference in the exploration of either object by either wild-type (*p* = 0.1536) of APP/PS1 mice (*p* = 0.5507; Fig. 2a) within the arena during the acquisition phase of the task. However, during the test phase of the novel ORT paradigm both wild-type and APP/PS1 murine groups showed significant preference for exploration of the ‘novel’ object (*p* < 0.05; Fig. 2b).

### APP/PS1 mice exhibit a low number of parenchymal thioflavin-S-positive plaques with no evidence of CAA at 4–5 months of age

**Fig. 3:**
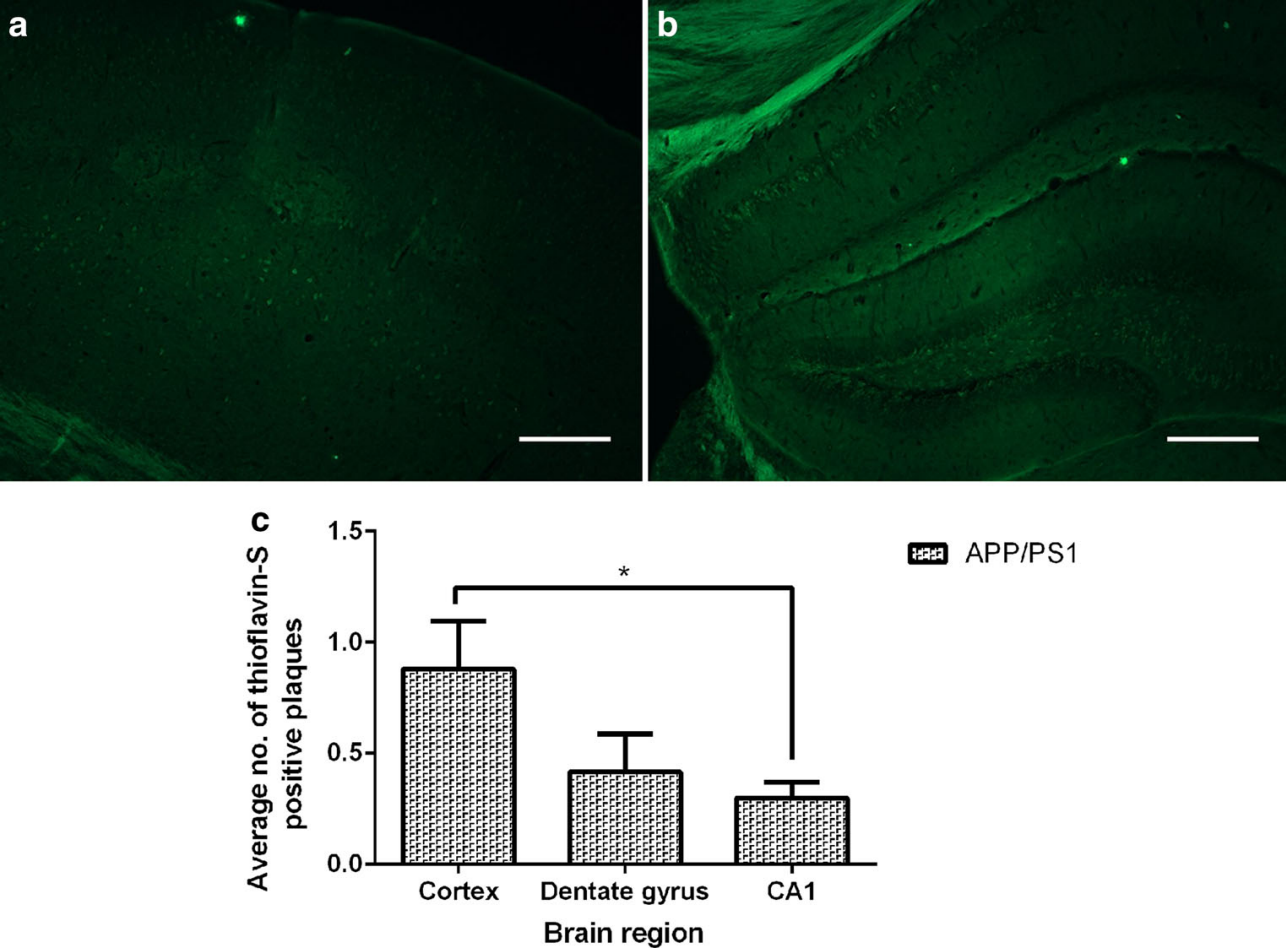
Low numbers of thioflavin-S-positive amyloid plaques were detected in both the cortex and hippocampus of 4- to 5-month-old APP/PS1 transgenic mice but not in their wild-type littermates. Representative fluorescent thioflavin-S stained micrographs of 20-μm-thick frozen brain sections showing single thioflavin-S stained plaques in the cortical (**a**) and hippocampal (**b**) brain regions from 4- to 5-month-old APP/PS1 mice; no thioflavin-S-positive plaques were observed in any of the brain sections examined in wild-type mice (data not shown). **c** The numbers of thioflavin-S plaques/field were significantly lower in the CA1 region than in the cortex (*p = 0.0152; *n* = 6 per group); all other statistical comparisons were non-significant (*p* > 0.05). Statistical analyses were conducted using either a Mann–Whitney test or unpaired *t* test and presented as mean ± SEM. *Scale bar* 200 μm

Examination via fluorescence microscopy of 20-μm-thick frozen sections of whole brain from 4- to 5-month-old wild-type mice (*n* = 6 per group) stained with thioflavin-S revealed there were no thioflavin-S-positive plaques in either the cortex or hippocampus (data not shown). There were a low number of thioflavin-S-positive plaques in the cortex (Fig. 3a) and the hippocampus (Fig. 3b) in age-matched 4- to 5-month-old APP/PS1 mice (*n* = 6 per group), in the absence of CAA. The number of thioflavin-S-positive plaques was significantly lower in the CA1 sub-region of the hippocampus when compared to the cortex of the 4- to 5-month-old APP/PS1 mice (*p = 0.0152; Fig. 3c). There was no significant difference in the number of plaques in the cortex and dentate gyrus (*p* = 0.1254) or between either region of the hippocampus (*p* = 0.5375; Fig. 3c).

### Multiple ultrastructural impairments in the cerebral cortical capillaries of 4- to 5-month-old APP/PS1 mice

**Fig. 4:**
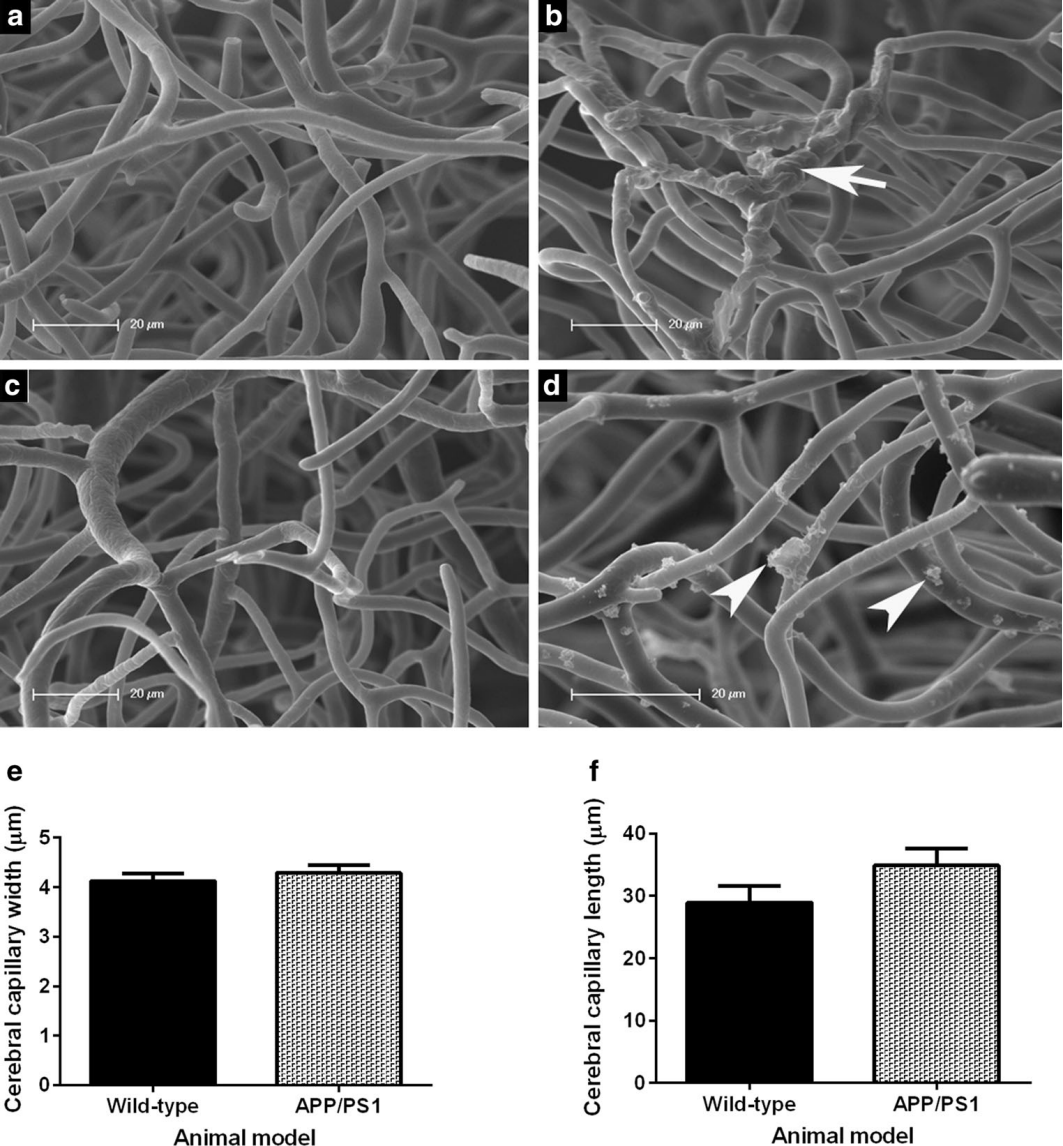
Representative scanning electron micrographs of resin-casted cortical vasculature within the brain of 4- to 5-month-old wild-type (**a**, **c**) and aged-matched APP/PS1 transgenic (**b**, **d**) mice (*n* = 6 per group). The intricate network of the cerebral cortical vessels of wild-type mice appears to have smooth lumenal profiles (**a**, **c**) in contrast to the cerebral vessels of the age-matched APP/PS1 mice that exhibit multiple microaneurysms along their lengths (*white arrow*; **b**) or across bifurcation points. Also, variously shaped resin extravasations lying orthogonal to the capillary axes remain attached to the vessel wall (*white arrowheads*; **d**). The average cortical capillary dimensions were not significantly different between groups (*p* = 0.4397; **e**) and (*p* = 0.1259; **f**). Statistical assessment was conducted using an unpaired *t* test and data are presented as mean ± SEM. *Scale bar* 20 µm

An ultrastructural examination of resin-casted cortical capillaries in the brain of 4- to 5-month-old wild-type mice revealed a regularly spaced network of interconnected capillaries with uniformly smooth lumenal surfaces (Fig. 4a, c). The resin-casted cerebral cortical capillaries of 4- to 5-month-old APP/PS1 mice exhibited microaneurysms both within individual vessel segments as well as spanning bifurcations. There were numerous and variously shaped resin protrusions (Fig. 4b; white arrow) conspicuous on discrete sections of capillary walls (Fig. 4d; white arrowheads). There were no significant differences in capillary width (*p* = 0.4397; Fig. 4e) or average cortical capillaries length (*p* = 0.1259; Fig. 4f) of the 4- to 5-month-old wild-type when compared to age-matched APP/PS1 transgenic mice.

### Highly significant reduction in the number of hepatic sinusoidal vessels on the serosal surface of the left hepatic lobe of 4- to 5-month-old APP/PS1 mice

**Fig. 5:**
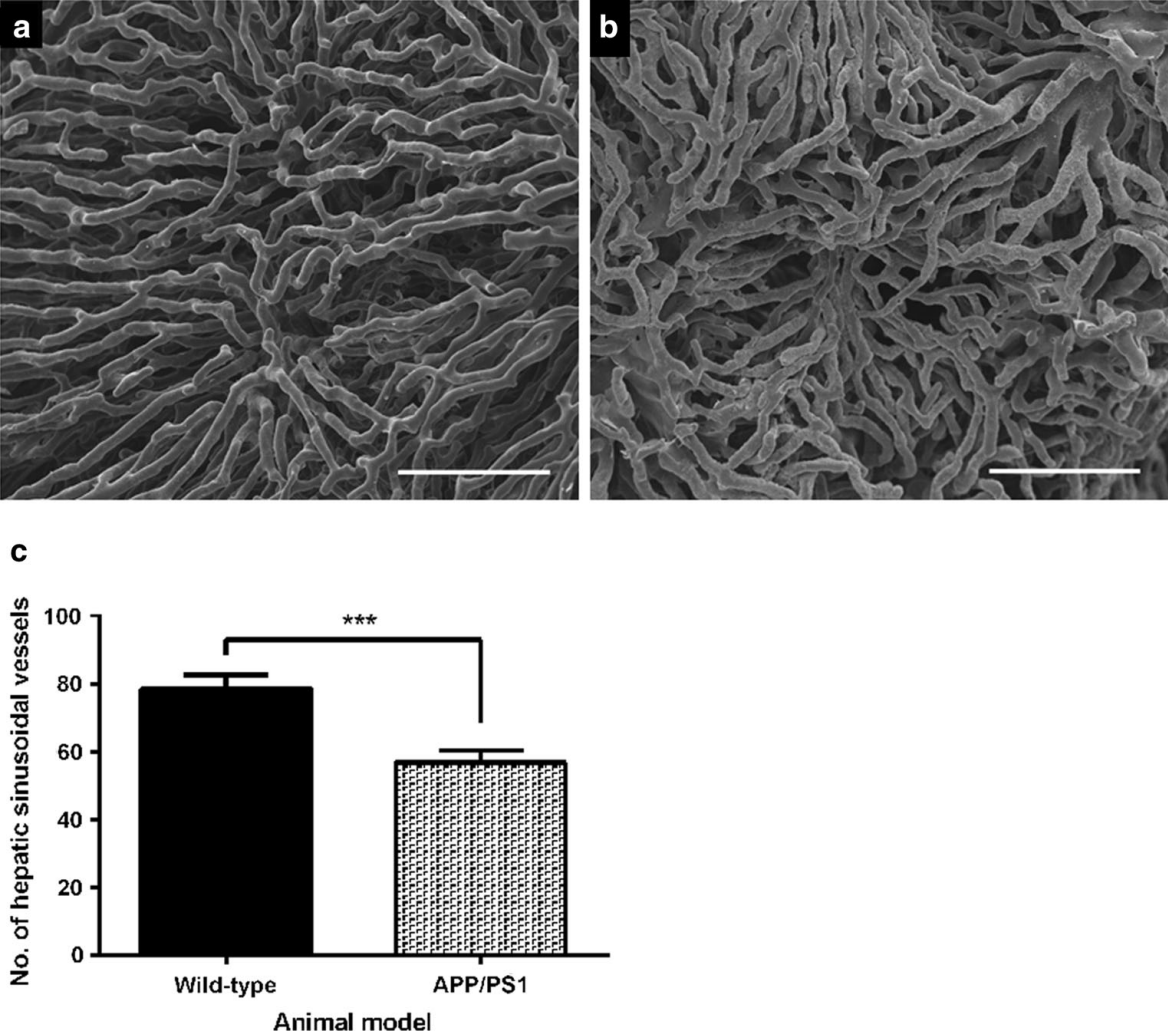
Representative scanning electron micrographs of resin-casted hepatic vasculature of the *left* hepatic lobe within 4- to 5-month-old wild-type (**a**) or APP/PS1 (**b**) mice (*n* = 6 per group). The hepatic angioarchitecture on the serosal surface of the left lobe consists of a network of interconnected vessels that have uniform diameters (**a**). The hepatic vasculature on the left lobe serosal surface of age-matched APP/PS1 mice contained areas of vessel elimination that were surrounded by densely crowded vessels (**b**). A quantitative evaluation of murine hepatic vessel number revealed a highly significant reduction in the number of hepatic sinusoidal vessels in APP/PS1 transgenic mice when compared to age-matched wild-type littermates (***p = 0.0002; **c**). Statistical analysis was conducted using an unpaired *t* test and data are presented as mean ± SEM. *Scale bar* 100 µm

The microvasculature on the serosal surface of the hepatic left lobe of 4- to 5-month-old wild-type mice contains slender sinusoidal vessels with uniform diameters that form a characteristic interconnecting pattern (Fig. 5a). The resin-casted hepatic vessels on the surface of the left hepatic lobe of age-matched APP/PS1 mice had gaps in the network adjacent to areas of more densely packed capillaries (Fig. 5b). Quantitation of hepatic vessel number on the hepatic serosal surface revealed a highly significant reduction in the number of microvessels in 4- to 5-month-old APP/PS1 when compared to age-matched wild-type mice (***p = 0.0002; Fig. 5c).

### Aberrant splenic vascular morphology and increased intussusception within the splenic sinuses of 4- to 5-month-old APP/PS1 mice

**Fig. 6:**
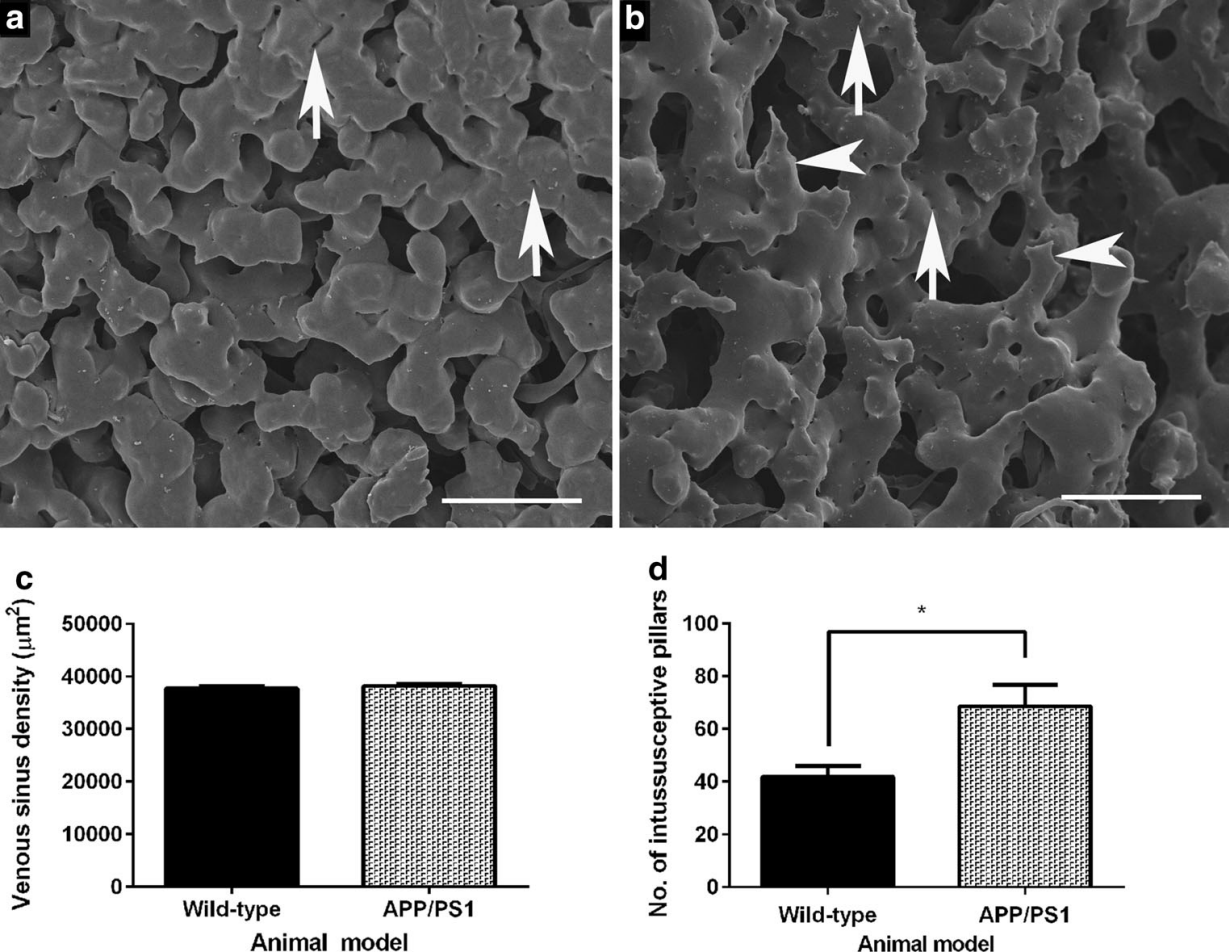
Representative scanning electron micrographs showing resin-casted vasculature on the serosal surface of the spleen of 4- to 5-month-old wild-type (**a**) and age-matched APP/PS1 (**b**) mice (*n* = 6 per group). The serosal surface of the murine spleen is covered with densely venous sinuses that contain differently sized intussusceptive pillars (*white arrow*; **a**). The venous sinuses on the splenic serosal surface of APP/PS1 transgenic mice appear irregular (*white arrowheads*; **b**) and also contain intussusceptive pillars (*white arrows*; **b**). There was no significant difference in splenic microvascular density between the murine groups (*p* = 0.3746; **c**); however, there was a significant increase in the number of intussusceptive pillars in the splenic microvasculature of 4- to 5-month-old APP/PS1 mice when compared to age-matched wild-type mice (*p = 0.0231; **d**). Statistical assessment was conducted using either Mann–Whitney or unpaired *t* test. Data are presented mean ± SEM. *Scale bar* 50 μm

An ultrastructural examination of the serosal surface of the spleen of 4- to 5-month-old wild-type mice (Fig. 6a) revealed a dense network of smoothly contoured bulbous venous sinuses containing intraluminal pillars (white arrows) that are a characteristic feature of the non-sprouting angiogenic process known as intussusceptive microvascular growth. In contrast, the splenic venous sinuses of age-matched APP/PS1 mice are thinner and have ragged-edges (white arrowheads) compared to those of wild-type mice (*cf*. Figure 6a, b). A quantitative evaluation of murine splenic vascular ultrastructure revealed no significant difference in splenic vascular density (*p* = 0.3746; Fig. 6c); however, the number of intussusceptive pillars within the splenic vasculature was significantly greater in APP/PS1 mice (*p* = 0.0231; Fig. 6d).

### Unremarkable glomerular vascular ultrastructure is associated with a highly significant reduction in uACR in 4- to 5-month-old APP/PS1 mice

**Fig. 7:**
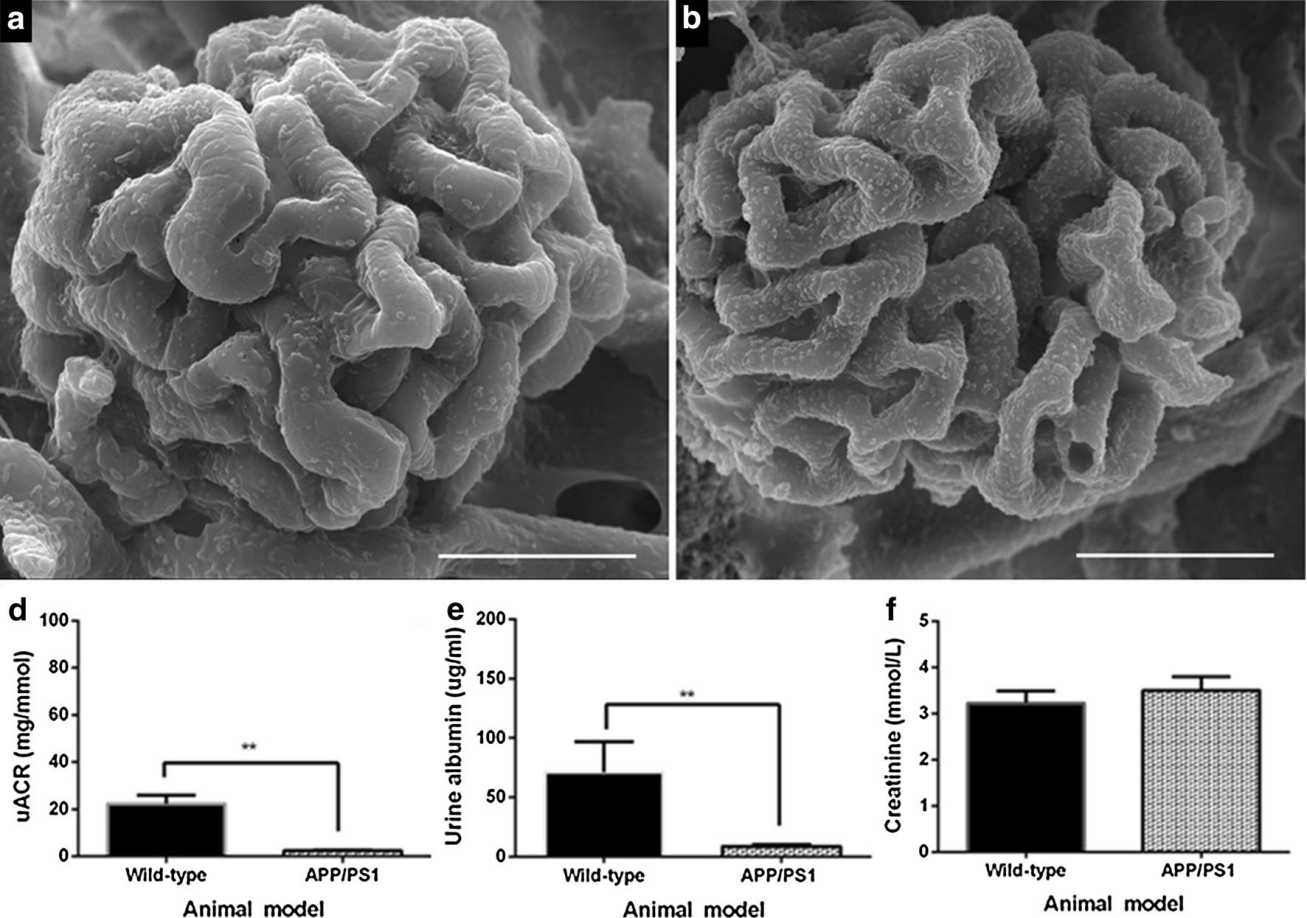
Representative scanning electron micrographs of murine glomerular microvasculature from 4- to 5-month-old wild-type (**a**) and APP/PS1 transgenic (**b**) mice (*n* = 6 per group) showing similar glomerular capillary profiles in both murine groups. A quantitative determination of the uACR in collected urine from 4- to 5-month-old wild-type and APP/PS1 (**c**–**e**) mice (*n* = 5 per group) revealed a significant reduction in uACR in APP/PS1 mice (**p = 0.0079; **c**) associated with a reduction in albumin excretion (**p = 0.0079; **d**) but not creatinine clearance (*p* = 0.4902; **e**) when compared to age-matched wild-type mice. Statistical evaluation conducted using either Mann–Whitney test or unpaired *t* test. Data are presented as mean ± SEM. *Scale bar* 20 µm

Scanning electron microscopic examination of resin-casted replicas from sagittally bisected mouse kidneys revealed that the glomerular capillary profiles of 4- to 5-month-old wild-type (Fig. 7a) and APP/PS1 mice (Fig. 7b) were ultrastructurally indistinguishable. However, a quantitative evaluation of collected mouse urine revealed a highly significant reduction in uACR in 4- to 5-month-old APP/PS1 mice when compared to age-matched wild-type mice (**p = 0.0079; Fig. 7c). In addition, there was also a highly significant reduction in the urinary albumin concentration in APP/PS1 mice when compared to the age-matched wild-type mice (**p = 0.0079; Fig. 7d). However, the urinary creatinine levels were not significantly different between groups (*p* = 0.4902; Fig. 7e).

## Discussion

Our previous work revealed multiple cerebral microvascular ultrastructural impairments in 9-month-old APP/PS1 mice [31] that coexist with AD neuropathologies, namely cerebral amyloid plaque burden, neuroinflammation and cognitive impairment [32]. Post-mortem studies of human AD brain tissues have previously reported concomitant microvascular ultrastructural changes that include atrophic and tortuous vessels in addition to the thickening of the microvascular basement membrane [41–43]. The aim of our present study was to quantitatively assess the fine structure of the vasculature in young 4- to 5-month-old APP/PS1 mice at an age that is prior to the onset of cognitive impairment in this murine model to determine whether pathological changes to the vasculature in the brain and peripheral organs are present.

The spontaneous locomotion and exploratory behaviour of 4- to 5-month-old wild-type and APP/PS1 transgenic mice was assessed using the OFT paradigm (Fig. 1). The 4- to 5-month-old APP/PS1 mice were more active within the open field than age-matched wild-type mice (Fig. 1a, b). This is consistent with a previous OFT study with cohorts of APP/PS1 mice that were 8- or 15-month-old that reported both age groups travelled a greater distance within the open field area than age-matched wild-type mice [44]. The increased activity exhibited by APP/PS1 mice within the arena may not be a result of increased anxiety which is associated with increased defecation and thigmotaxis during the OFT [44, 45]. Our findings show that APP/PS1 mice exhibited no significant preference in the exploration of the arena and there were no significant differences in the number of faecal pellets produced by APP/PS1 mice during the task when compared to age-matched wild-type mice.

The APP/PS1 transgenic mice have been reported to have an intact recognition memory at 6 months of age [46] with deficits in spatial memory detectable from 7 months of age [30, 32, 44]. Accordingly, we examined the working memory of 4- to 5-month-old mice within the novel ORT paradigm (Fig. 2) and found that both groups of mice showed a significant preference for the exploration of an object not previously encountered during the acquisition phase of the trial (Fig. 2b). This finding suggests that the recognition memory of 4- to 5-month-old wild-type and APP/PS1 mice are intact and that 4- to 5-month-old APP/PS1 mice represent a time point in the pathogenesis of AD in this murine model that is prior to the onset of cognitive impairment.

The cerebral amyloid plaque burden in 4- to 5-month-old mice was fluorescently determined using the methylated primuline, thioflavin-S [47] histological stain, which competes for the same binding site on amyloid plaques as the widely used Congo red stain [48]. We found no parenchymal or vascular thioflavin-S-positive plaques in either the cortex or hippocampus of 4- to 5-month-old wild-type mice, whereas the age-matched APP/PS1 mice had a very low number of plaques in the cortex and hippocampus in the absence of CAA (Fig. 3), which is consistent with previous reports [28, 49]. The APP/PS1 transgenic murine model of AD does progressively accumulate cerebral amyloid plaque deposits in the cortex and hippocampus brain region between the ages of 4–12 months of age [28]. A similar cortical and hippocampal specific deposition of amyloid plaques has been described in human brain tissues [50].

We replicated the cerebrovasculature of 4- to 5-month-old mice using the microvascular corrosion casting technique with a polyurethane-based PU4ii casting resin (Fig. 4) that had favourable physical characteristics such as a low viscosity, elasticity and minimal shrinkage [51]. The resin-casted murine cerebral capillaries we recreated and observed by SEM had an average diameter of 4.1 µm in wild-type mice and 4.3 µm in APP/PS1 mice (Fig. 4e), which is similar to the baseline capillary diameters of 4.4 µm previously reported in anaesthetised C57BL/6J mice [52]. This supports an accurate replication of the murine cerebral capillary network using the microvascular corrosion casting technique.

We consistently observed multiple clustered resin extravasations (of <5 µm in length) attached to cerebral capillary lumens within brains of 4- to 5-month-old APP/PS1 mice but not age-matched wild-type animals (Fig. 4b, d). These structures suggest a breakdown of the BBB in 4- to 5-month-old APP/PS1 mice, which is prior to the onset of cognitive impairment in this murine AD model. The casting resin solution may have leaked through or between adjacent endothelial cells during its polymerisation within the vessel wall due to an aberrant increase in the permeability of the cerebral microvessels in 4- to 5-month-old APP/PS1 mice. An increased cerebrovascular permeability has been described previously in 9-month-old APP/PS1 mice using corrosion casting with SEM [31] and 12-month-old APP/PS1 mice via electron microscopy and use of an intravenously administered Evans-blue dye [53] as well as in cohorts of 14-month-old or 24-month-old APP/PS1 mice using MRI detection of a gadolinium-based contrast agent [54]. Furthermore, an age-dependent pericyte-loss in murine models of deficient platelet-derived growth factor receptor beta (PDGFR-β) signalling showed cerebrovascular degeneration, increased BBB permeability with an unregulated brain entry and perivascular accumulation of serum-derived proteins preceded and most likely contributed to the secondary degenerative changes in neuronal structure, circuitry and behaviour in the novel ORT paradigm [55].

Our previous published findings describe the microvascular ultrastructural impairments in the spleen and kidneys of 9-month-old APP/PS1 mice. We showed that 9-month-old APP/PS1 mice have a significant reduction in splenic microvascular density and exhibit ultrastructural impairments in glomerular capillaries [31]. The findings of our present study show microvascular ultrastructural impairments in the liver, spleen and kidneys of APP/PS1 mice at 4–5 months of age. This is particularly relevant as the intravenous administration of a ^125^I-Aβ-40 tracer to wild-type and APP/PS1 mice showed that the peripheral clearance of Aβ-40 peptide occurred via the liver, spleen and kidneys [56]. Here we observed a highly significant reduction in the number of sinusoidal vessels on the serosal surface of the left hepatic lobe of 4- to 5-month-APP/PS1 mice when compared to age-matched wild-type mice (Fig. 5). Interestingly, findings of a previous study showed that the perfusion on the surface of left hepatic lobe of C56BL/6 mice was reduced by 35% between the ages of 0.8–24 months of age [57]. Furthermore, ageing-related changes to hepatic sinusoidal vessels such as loss of fenestration and endothelial thickening have been described in human liver specimens at post-mortem [58]. Additionally, ligation of the portal vein and coeliac artery in rats was shown to impede the cerebral clearance of Aβ 1–40 peptide [59], suggesting that the aberrant hepatic structural changes we observed in 4- to 5-month-old APP/PS1 mice could negatively impact upon the cerebral clearance of Aβ 1–40 peptide, which could promote amyloid plaque burden and CAA.

We observed aberrant venous sinus morphology in the splenic microvasculature of 4- to 5-month-old APP/PS1 mice when compared to age-matched wild-type mice (Fig. 6). The splenic microvascular changes were coincident with an increased number of intussusceptive pillars that are distinctive features of ‘in-itself’ microvascular growth, first observed within casted replicas of vasculature within rat lung [60, 61]. In contrast to classical sprouting angiogenesis, intussusceptive angiogenesis (IA) is energy efficient, faster and involves the formation of intraluminal pillars that fuse together to split an existing vessel into two vessels [62]. IA is a ubiquitous process which has been proposed to be regulated by microhemodynamics [63] and previously reported to occur in the vasculature of rats, mice and humans [64]. Intussusceptive angiogenesis has also been observed in murine models of colitis, in which pillars are observed as ‘pits’ in corrosion casts of the mucosal plexus at 4–7 days following chemical induction of colitis, as an adaptive response to heightened metabolic demand due to the onset of inflammation [63]. Interestingly, these authors report an intussusceptive-mediated remodelling of the entire mucosal plexus by 4 weeks, attributed to enhanced survival of this murine model [63]. We have also previously shown, by SEM of vascular casts, that there is also a 30% reduction in splenic microvasculature in 9-month-old APP/PS1 transgenic mice as compared to age-matched wild-type mice [31]. Taken together, these data support the assertion that the significant increase in intussusceptive pillars in the spleen at 4–5 months of age reflects an early splenic hemodynamic compromise, which leads to the more substantial vessel loss observed in 9-month-old APP/PS1 mice.

We observed no striking differences in murine glomerular capillary morphology in APP/PS1 mice compared to wild-type mice (Fig. 7). However, we identified a highly significant and consistent reduction in the uACR of 4- to 5-month-old APP/PS1 mice, compared to wild-type mice (Fig. 7c). The findings of our present study indicate an aberrant functioning in renal albumin excretion, prior to the onset of cognitive deficits in young 4- to 5-month-old APP/PS1, warranting a further examination of glomerular capillary structure and function in young APP/PS1 mice. Interestingly, a nuclear magnetic resonance metabolomic study of 4-month-old APPswe/tau AD mice [65] revealed elevated markers of oxidative stress in urine, which similar to our study, is also prior to the onset of cognitive impairment in this murine model [65]. Furthermore, a study of >2100 individuals with a mean age of 56 years old reported there to be a strong association between a reduction in the glomerular filtration rate and the prevalence of white matter hyperintensities (WMH), observed during MRI, which were reported to be independent of hypertension [66]. WMH include the demyelination of axons, tissue rare fraction and gliosis and were reported in a meta-analysis of longitudinal MRI studies to be predictive of an increased rate of cognitive decline [67].

Taken together, the findings of our study, in young cognitively healthy 4- to 5-month-old APP/PS1 mice with a low number of parenchymal amyloid plaques with no CAA, reveal early pathological changes to the cerebral cortical capillaries, hepatic sinusoidal vessels, splenic venous sinuses in addition to impairments in renal albumin clearance that may be early indicators of progression to severe disease in this experimental AD murine model. These findings warrant further examination of the role that cerebral and systemic microvascular pathologies play in the progression of neurodegeneration to determine whether the vasculature is a diagnostic target with prophylactic efficacy in slowing neurodegeneration.
